# Annual acknowledgement of manuscript reviewers

**DOI:** 10.1186/1471-2229-14-30

**Published:** 2014-01-18

**Authors:** Simon Harold

**Affiliations:** 1 BioMed Central, Floor 6, 236 Grey's Inn Road, London WC1X 8HB, UK

## Abstract

**Contributing reviewers:**

The editors of BMC Plant Biology would like to thank all of our reviewers who have contributed to the journal in Volume 13 (2013).

## 

Ibrokhim Abdurakhmonov

Uzbekistan

Amanda Able

Australia

Sue Abrams

Canada

Araceli Aguilar-Melendez

Mexico

pmvir Ahuja

India

Kazuhito Akama

Japan

Jose A Alcalde

Chile

Andrew Allan

New Zealand

Sara Amancio

Portugal

Jeppe R Andersen

Denmark

Charles Anderson

USA

Limami Anis

France

Koh Aoki

Japan

Felipe Aquea

Chile

Maria Jose Aranzana

Spain

Catalina Arenas-Huertero

Mexico

Lirio Arevalo-Soliz

USA

Hamed Arisha

Egypt

Mario Arteaga-Vazquez

Mexico

Robert Augustine

USA

Belay Ayele

Canada

Brian Ayre

USA

Driouich Azeddine

France

Ivan Baccelli

Italy

Christian Bachem

Netherlands

Charles Bacon

USA

Shunong Bai

China

Yuling Bai

Netherlands

Julia Bailey-Serres

USA

Sureshkumar Balasubramanian

Australia

Elizabeth Baldwin

USA

Dirk Balmer

Switzerland

Valeria Banti

Italy

Todd Barkman

USA

Amalia Barone

Italy

Celia Baroux

Switzerland

Blanca Estela Barrera

Mexico

Craig Barrett

USA

Yves Barriere

France

Diane Bassham

USA

Daniele Bassi

Italy

Roberto Bassi

Italy

Nahla Bassil

USA

Henri Batoko

Belgium

Stefan Bauer

USA

Gregory Baute

Canada

Nathalie Beaudoin

Canada

Ulrike Bechtold

UK

Jules Beekwilder

Netherlands

Eric Beers

USA

Faith Belanger

USA

Jayne Belnap

USA

Gary Bending

UK

Ana Maria Benko-Iseppon

Brazil

Dave Berger

South Africa

Fred Berger

Singapore

Jamila Bernardi

Italy

Pankaj Bhardwaj

India

Prasanna Bhat

India

Mrinal Bhave

Australia

Gerd Patrick Bienert

Germany

K Bilyeu

USA

Brad Binder

USA

Stefania Biondi

Italy

Craita E Bita

Netherlands

Benjamin Blonder

USA

Arnold Bloom

USA

Thomas Boerner

Germany

Christine Boettcher

Australia

Laszlo Bogre

UK

Melvin Bolton

USA

Pierluigi Bonello

USA

Claudio Bonghi

Italy

Guusje Bonnema

Netherlands

Maria Borisova-Mubarakshina

Russian Federation

Andrew Bottley

UK

Alessandro Botton

Italy

Adnane Boualem

France

Nicolas Bouche

France

John Bowman

Australia

Andrea Braeutigam

Germany

Katharina Braeutigam

Canada

Federica Brandizzi

USA

Thomas Braukmann

Canada

David Braun

USA

Patrick Brown

USA

Stevens Brumbley

USA

Teerapong Buaboocha

Thailand

C. Robin Buell

USA

Lorenzo Burgos

Spain

Daniel Bush

USA

John Bussell

Australia

Melinka Butenko

Norway

Ivan Maureira Butler

Chile

Catalina Cabot

Spain

Mario Caccamo

UK

Pat Calie

USA

Ann Callahan

USA

Myriam Calonje

Spain

Maura Cannon

USA

Daniela Capiati

Argentina

Pablo Carbonell-Bejerano

Spain

Francesca Cardinale

Italy

Maria José Carmona

Spain

John Peter Carr

UK

Clay Carter

USA

Jorge Casal

Argentina

Simone Castellarin

Italy

Eduardo A Ceccarelli

Argentina

Mariateresa Cervera

Spain

David Chagne

New Zealand

Jordi Chan

UK

Zhulong Chan

China

Kent Chapman

USA

Yee-Yung Charng

Taiwan

Christine Chase

USA

Feng Chen

USA

Kun-Song Chen

China

Rujin Chen

USA

Shilin Chen

China

Xianming Chen

USA

Xiao-Ya Chen

China

Xinbo Chen

China

Yi Chen

UK

Choong-Ill Cheon

Korea, South

Christian Chervin

France

Yukako Chiba

Japan

Maria Luisa Chiusano

Italy

Hye Sun Cho

Korea, South

Kang Chong

China

Zamioudis Christos

Netherlands

Ye Chu

USA

Stephan Clemens

Germany

Sylvie Cloutier

Canada

Jeremy Coate

USA

Elroy Cober

Canada

Enrique Cointry

Argentina

Luca Comai

USA

Emily Combs

USA

Eleonora Cominelli

Italy

David Cook

USA

Janice Cooke

Canada

Andrew Cooper

USA

Gregory Copenhaver

USA

Sylvain Cordelier

France

Dorina Creanga

Romania

Hong Cui

China

James Culver

USA

Yang Cunyi

China

Stefan Czemmel

USA

Luca Dall'Osto

Italy

Tamas Dalmay

UK

Grimanelli Daniel

France

Karine David

New Zealand

Christopher Davies

Australia

Mike Davis

USA

Seth J Davis

Germany

Stefan De Folter

Mexico

Barend De Graaf

UK

Bert De Rybel

Netherlands

Ahmed Debez

Germany

John Paul Delano-Frier

Mexico

Antonio Delgado

Spain

Alfonso Delgado-Salinas

Mexico

Manny Delhaize

Australia

Massimo Delledonne

Italy

Serge Delrot

France

Michel Delseny

France

Laurent Deluc

USA

Hai Hua Deng

China

Dorine Desalme

France

Monique Deu

France

Michael K Deyholos

Canada

Christophe D'Hulst

France

Rebecca Dickstein

USA

Stephen Difazio

USA

Paul Dijkwel

New Zealand

Christine Dillmann

France

Tzvetanka Dimitrova Dinkova

UK

Franck Ditengou

Germany

Ian Dodd

UK

Colleen Doherty

USA

Jaroslav Dolezel

Czech Republic

Kara Dolinski

USA

Kevin Dorn

USA

Mark Downey

Australia

Jeff Doyle

USA

Kurt Drickamer

UK

Ian Dry

Australia

Eric Duchene

France

Stefanie Dukowic-Schulze

USA

Frank Dunemann

Germany

Peter Eastmond

UK

Atsushi Ebihara

Japan

Wolfgang Ecke

Germany

Ashley N Egan

USA

Marcos Egea-Cortines

Spain

Ulrika Egertsdotter

USA

Frank Eisenhaber

Singapore

Carolina Escobar

Spain

Richard Espley

New Zealand

Gonzalo Estavillo

Australia

Jose Estevez

Argentina

Thomas Eulgem

USA

Daniele Evers

Luxembourg

Fabio Fabio

Italy

Ahmed Faik

USA

Patricia Faivre Rampant

France

Chuanzhu Fan

USA

Edward Farmer

Switzerland

Zhangjun Fei

USA

Albert Ferrer

Spain

Janette Palma Fett

Brazil

Ivo Feussner

Germany

Antonio Figueira

Brazil

Elizabeth Jean Finnegan

Australia

Iris Fischer

France

Teresa Fitzpatrick

Switzerland

Sherry Flint-Garcia

USA

Francisco Florencio

Spain

Victor Flors

Spain

Jonathan Flowers

USA

Jeffrey Foster

USA

Vasileios Fotopoulos

Cyprus

Claudine Franche

France

Robert Franks

USA

Elisabetta Frascaroli

Italy

John Freeman

USA

Margrit Frentzen

Germany

Wieland Fricke

Ireland

Eyal Fridman

Israel

Lutz Froenicke

USA

Florian Frugier

France

Julia Frugoli

USA

Yong-Fu Fu

China

Karen Fugate

USA

Kenji Fujino

Japan

Toru Fujiwara

Japan

Atsushi Fukushima

Japan

Leonardo Galetto

Argentina

Gabor Galiba

Hungary

Giulio Galla

Italy

Pooba Ganeshan

Canada

Caiji Gao

Hong Kong

Dongying Gao

USA

Shumin Gao

China

Zhong-Shan Gao

China

Adriana Garay

Mexico

Rohini Garg

India

Sonia Gazzarrini

Canada

Anja Geitmann

Canada

Jonathan Gent

USA

Rene Geurts

Netherlands

Koen Geuten

Belgium

Godelieve Gheysen

Belgium

Glenda Gillaspy

USA

Jason Gillman

USA

Mike Giroux

USA

Giovanni Giuliano

Italy

Heiner Goldbach

Germany

John Golz

Australia

Aurelio Gomez-Cadenas

Spain

Enrique Gonzalez

Chile

Reimund Goss

Germany

Charles Goulet

Canada

Udo Gowik

Germany

Gideon Grafi

Israel

Lydia Gramzow

Germany

Katja Graumann

UK

John Gray

USA

Per Gregersen

Denmark

Brian Gregory

USA

Stephan Greiner

Germany

Christopher Grof

Australia

Kristina Gruden

Slovenia

Rebecca Grumet

USA

Gea Guerriero

Luxembourg

Mei Guo

USA

Yongfeng Guo

China

Yufang Guo

USA

Michael Gutensohn

USA

Charles Guy

USA

Flavia Guzzo

Italy

Christine Hackett

UK

Nian Hai

China

Candace Haigler

USA

Fengxiang Han

USA

Oksoo Han

Korea, South

Yuko Hanba

Japan

Kerry Hancock

New Zealand

Thomas James Hardcastle

UK

Bettina Hause

Germany

Zoltan Havelda

Hungary

Michael Havey

USA

Christopher Hawes

UK

Samuel Hazen

USA

Hang He

China

Sheng Yang He

USA

Xinqiang He

China

Yuke He

China

Peter Hedden

UK

Alain Hehn

France

Roger Paul Hellens

New Zealand

Chris Helliwell

Australia

Vera Hemleben

Germany

Lars Hennig

Switzerland

Holger Hesse

Germany

Tarek Hewezi

USA

Julian Hibberd

UK

Imene Hichri

Belgium

David Hildebrand

USA

Candice Hirsch

USA

Frank Hochholdinger

Germany

Owen Hoekenga

USA

Monica Hofte

Belgium

Noel Holbrook

USA

David Holding

USA

Daniela Holtgraewe

Germany

Chwan-Yang Hong

Taiwan

Csaba Hornyik

UK

Stefan Hortensteiner

Switzerland

Catherine Howarth

UK

Rongfeng Huang

China

Tengfang Huang

USA

Karen Hudson

USA

Arthur Hunt

USA

Donald Hunter

New Zealand

Torgeir R Hvidsten

Norway

Timo Hytönen

Finland

Richard Immink

Netherlands

Hideyuki Inui

Japan

Muhammad Iqbal

USA

Tal Isaacson

Israel

Abdelbagi Ismail

Philippines

Yasuhiro Ito

Japan

Takeshi Itoh

Japan

Akira Iwase

Japan

Scott Jackson

USA

Thomas Jacobs

USA

Mukesh Jain

India

Pankaj Jaiswal

USA

Gabor Jakab

Hungary

Tibor Janda

Hungary

Malgorzata Janicka-Russak

Poland

Artur Jarmolowski

Poland

Paul Jarvis

UK

Matthew Jenks

USA

Bhavanath Jha

India

Ning Jiang

USA

Yuling Jiao

China

Hai-Chun Jing

China

Udo Johanningmeier

Germany

Linda Johnson

New Zealand

Veronique Jorge

France

Ji Hyung Jun

USA

Tetsuji Kakutani

Japan

Udaya Kalluri

USA

Byung-Ho Kang

USA

Sirpa Kärenlampi

Finland

Rumyana Karlova

Netherlands

Kerstin Kaufmann

Germany

Yoji Kawano

Japan

Kemal Kazan

Australia

Beat Keller

Switzerland

Stefan Kempa

Germany

Sekine Ken-Taro

Japan

Bouchaib Khadari

France

Khalid Mahmud Khokhar

Pakistan

Dominikus Kittemann

Germany

Christian Klukas

Germany

Hanna Kmita

Poland

Volker Knoop

Germany

Tobias Koellner

Germany

Claudia Kohler

Sweden

Yoshibumi Komeda

Japan

Maarten Koornneef

Germany

Ian Korf

USA

Jens Kossmann

South Africa

Franziska Krajinski

Germany

Brian Kram

USA

John Kress

USA

Beth Krizek

USA

Pawel Krupinski

Sweden

Ajay Kumar

USA

Vasu Kuraparthy

USA

Leon Kurepin

Canada

June Kwak

USA

Pieter Bas Kwak

USA

Maite Lacuesta

Spain

Zhibing Lai

USA

Eric Lam

USA

Sheung Kwan Lam

USA

Derek Thomas Anthony Lamport

UK

Bernd Markus Lange

USA

Maria Valeria Lara

Argentina

André Laroche

Canada

Patrick Laufs

France

Hernan Laurentin

Venezuela

Thomas Laux

Germany

Matt Lavin

USA

Susan Lawrence

USA

Fatma Lecourieux

France

David Lee

UK

Carolyn Lee-Parsons

USA

Valerie Legué

France

Andrew Leitch

UK

Krzysztof Lesniewicz

Poland

Antonio Leyva

Spain

Feng Li

China

Ling Li

USA

Qing Li

USA

Rugang Li

USA

Xuehui Li

USA

Yangsheng Li

China

Yongchun Li

China

Zhenghe Li

China

Zhongyi Li

Australia

Verónica Lia

Argentina

Hong Liao

China

David Lightfoot

USA

Wilco Ligterink

Netherlands

Hongxuan Lin

China

Rongcheng Lin

China

Yao-Cheng Lin

Belgium

Steven Lindow

USA

Lucia Lioi

Italy

Vincenzo Lionetti

Italy

Chang-Jun Liu

USA

Chengke Liu

USA

Hongjun Liu

China

Hongtao Liu

China

Peng Liu

China

Qing-Lin Liu

China

Sanzhen Liu

USA

Yule Liu

China

Zhongchi Liu

USA

Maren Livaja

Germany

Wayne Loescher

USA

Maria Logacheva

Russian Federation

Yan Long

China

Damar Lizbeth Lopez-Arredondo

Mexico

Jorge Lora Cabrera

Spain

Yonggen Lou

China

John Love

UK

Meng-Zhu Lu

China

Pingli Lu

China

Shiyou Lu

China

Yanli Lu

China

Ndiko Ludidi

South Africa

Joost Luecker

Australia

Michelle Lum

USA

Estrella Luna Diez

UK

Elisabetta Lupotto

Italy

Qing-Hu Ma

China

Ramamurthy Mahalingam

USA

Manoj Majee

India

George Manganaris

Cyprus

Nitin Mantri

Australia

Annie Marion-Poll

France

Daniela Marone

Italy

Stefan Martens

Italy

Rebeca Martinez

Canada

Angel Emilio Martinez De Alba

France

Kyonoshin Maruyama

Japan

Martin Mascher

Germany

Hugh Mason

USA

Kenji Matsui

Japan

Autar Mattoo

USA

Tim Mauchline

UK

Veronica Maurino

Germany

Phillip Mcclean

USA

C. Robertson McClung

USA

Heather Mcfarlane

Germany

J. Mitchell McGrath

USA

Mitch Mcgrath

USA

Cecilia Mcgregor

USA

Leah Mchale

USA

Joel Mcneal

USA

Tim Mcnellis

USA

Kevin Mcphee

USA

F. Javier Medina

Spain

Andy Meharg

UK

Patrice Meimoun

France

Rainer Melzer

Germany

Johan Memelink

Netherlands

Venugopal Mendu

USA

Kristin Mercer

USA

Ebe Merilo

Estonia

Scott Merkle

USA

Mark Mescher

USA

Rainer Messmer

Switzerland

Peter Meyer

UK

Bruno Mezzetti

Italy

Yansong Miao

USA

Mariangela Miele

Italy

Subhash Minocha

USA

Amitava Mitra

USA

Birgit Mitter

Austria

Yutaka Miyazawa

Japan

Wolfgang Moeder

Canada

Athanassios Molassiotis

Greece

Antonio Molina

Spain

Ximena Moncada

Chile

Tapan Mondal

India

Antonio Jose Monforte

Spain

Josselin Montarry

France

Pascal Montoro

France

John Morgan

USA

Takaya Moriguchi

Japan

Tomas Morosinotto

Italy

Matthew Moscou

UK

Claudio Moser

Italy

Agnieszka Mostowska

Poland

Mario Motto

Italy

Zhonglin Mou

USA

Grégory Mouille

France

Gary Muehlbauer

USA

Lukas Mueller

USA

Karl Mühling

Germany

David Mulcahy

USA

Robert Mullen

Canada

Karel Müller

Czech Republic

Philip Mullineaux

UK

K Muramoto

Japan

Yoshiyuki Murata

Japan

Angus Murphy

USA

Alan Myers

USA

Jim Myers

USA

Sean Myles

Canada

Kirankumar Mysore

USA

Kiyotaka Nagaki

Japan

Kyoung Hee Nam

Korea, South

Judith Nardmann

Germany

Annette Nassuth

Canada

Lorella Navazio

Italy

Hilde Nelissen

Belgium

Rebecca Nelson

USA

Timothy Nelson

USA

Massimo Nepi

Italy

Alexandre Nepomuceno

USA

Jean-Marc Neuhaus

Switzerland

Godfrey Neutelings

France

Steven Newmaster

Canada

Zhongfu Ni

China

Paul Nicholson

UK

Peter Nick

Germany

Antoine Nicolas

USA

Erik Nielsen

Italy

Kazuhiko Nishitani

Japan

Graham Noctor

France

Ko Noguchi

Japan

Enrico Noli

Italy

Takeshi Obayashi

Japan

Alessandro Occhialini

UK

Remko Offringa

Netherlands

Yoshiyuki Ogata

Japan

Man-Ho Oh

USA

Akemi Ohmiya

Japan

Akio Ohyama

Japan

Akira Oikawa

Japan

Yozo Okazaki

Japan

M. Margarida Oliveira

Portugal

Enrique Olmos

Spain

Carmel O'Neill

UK

Malcolm O'Neill

USA

Lars Opgenoorth

Germany

David Oppenheimer

USA

Jamie O'Rourke

USA

Rodomiro Ortiz

Sweden

Takashi Osono

Japan

Peggy Ozias-Akins

USA

Sudheer Pamidimarri

India

Sona Pandey

USA

Florent Pantin

UK

Omar Pantoja

Mexico

Roberto Papa

Italy

Csaba Papdi

UK

Roger Parish

Australia

Christian Parisod

Switzerland

Phun Bum Park

South Korea

Sang-Wook Park

USA

John Patrick

Australia

Stefano Pavan

Japan

Tomasz Pawlowski

Poland

Mario Enrico Pè

Italy

Jean-Claude Pech

France

Pai Pedas

Denmark

Lola Peñarrubia

Spain

Liangcai Peng

China

Michele Perazzolli

Italy

Dragan Perovic

Germany

Staffan Persson

Germany

John Pickett

UK

Marco Pietrella

Italy

Pietro Piffanelli

Italy

Ken Piller

USA

Yves Poirier

Switzerland

Annalisa Polverari

Italy

Maharajah Ponnaiah

France

Ron Porat

Israel

Ezio Portis

Italy

Curtis Pozniak

Canada

Manoj Prasad

India

Ram Prasad

India

Gail Preston

UK

Jill Preston

USA

Sasha Preuss

USA

Humberto Prieto

Chile

Jaime Prohens

Spain

Holger Puchta

Germany

Liwang Qi

China

Yiping Qi

USA

Yongwen Qi

China

Feng Qin

China

Feng Qu

USA

Rongda Qu

USA

Elena Simona Radutoiu

Denmark

Ramesh Raina

USA

Istvan Rajcan

Canada

Harsh Raman

Australia

Christopher Randle

USA

Guang-Yuan Rao

China

Ryan Rapp

USA

Pascal Ratet

France

Animesh Ray

USA

Rob Reid

Australia

Susanne Renner

Germany

Stefan Rensing

Germany

Natalia Requena

Germany

Franko Restovic

Chile

Summaira Riaz

USA

Rosa M Rivero

Spain

Eric Roalson

USA

Alison Roberts

USA

Jeremy Roberts

UK

Mike Roberts

UK

Victor Rodriguez

Spain

Odd Arne Rognli

Norway

Charles Romieu

France

Fred Rook

Denmark

Tadeusz Rorat

Poland

Alan Rose

USA

Jocelyn Rose

USA

Rebecca Roston

USA

Giuseppe Leonardo Rotino

Italy

Kalliopi Roubelakis-Angelakis

Greece

Anne Roulin

Switzerland

Matthew Rouse

USA

Jean-Marc Routaboul

France

Jianyun Ruan

China

Paul Rushton

USA

Choong-Min Ryu

Korea, South

Atsushi Sakai

Japan

Yoh Sakuma

Japan

M. Teresa Sanchez-Ballesta

Spain

Ajay Sandhu

USA

Daniel Sargent

Italy

Daniel Sargent

UK

Mirella Sari Gorla

Italy

Ananda Sarkar

India

Scott Sattler

USA

Arnould Savoure

France

Shinichiro Sawa

Korea, North

Samir Sawant

India

Olga Sayanova

UK

Jan Schaart

Netherlands

Robert Schaffer

New Zealand

Brian Scheffler

USA

Sarah Schiessl

Germany

Stefan Schillberg

Germany

Jessica Schlueter

USA

Karl Schmid

Germany

James Schnable

USA

Gerald M Schneeweiss

Austria

Danny Schnell

USA

Henk Schouten

Netherlands

Lukas Schreiber

Germany

Ingo Schubert

Germany

Claus Schwechheimer

Germany

Wolfgang Schweiger

Austria

Jean-Paul Schwitzguebel

Switzerland

Steven Scofield

USA

Paul Scott

USA

Luca Sebastiani

Italy

John Sedbrook

USA

Vincent Segura

France

Champa Sengupta-Gopalan

USA

Yong Weon Seo

Korea, South

Kjell Sergeant

Luxembourg

Kiran Sharma

India

Ram Kumar Sharma

India

Peter Sharp

Australia

Jeff Shen

USA

Wenbiao Shen

China

Qinghua Shi

China

Ming-Der Shih

Taiwan

Tomoo Shimada

Japan

Jae-Ho Shin

Korea, South

Jin Hee Shin

USA

Jay Shockey

USA

Tsubasa Shoji

Japan

Huixia Shou

China

Allan Showalter

USA

Richard Sibout

France

Richard Sicher

USA

Lyudmila Sidorenko

UK

Narendra Singh

USA

Daniel Sloan

USA

Janet Slovin

USA

Jan Smalle

USA

Lawrence Smart

USA

Aaron Smith

USA

Alison Smith

UK

Penelope Smith

Australia

Imre Somssich

Germany

Kwan Jeong Song

Korea, South

Alvaro Soto

Spain

Francesca Sparvoli

Italy

Wolfgang Spielmeyer

Australia

Vibha Srivastava

USA

Claudia Stange

Chile

Giovanni Stefano

USA

Nils Stein

Germany

Lukasz Stelinski

USA

Egidio Stigliano

Italy

Maciej Stobiecki

Poland

Maria C Suarez Rodriguez

USA

Koichi Sugimoto

Japan

Akifumi Sugiyama

Japan

Serenella Sukno

Spain

Qi Sun

USA

Qixin Sun

China

Ramanjulu Sunkar

USA

Nada Surbanovski

Italy

Fedora Sutton

USA

Masashi Suzuki

Japan

Noriko Suzuki

Japan

Robert Swanson

USA

Olga Sztatelman

Poland

Daniel Szymanski

USA

Naoki Takata

Japan

Manuel Talon

Spain

Li Tan

USA

Tsuyoshi Tanaka

Japan

Georgia Tanou

Greece

Celine Tasset

Australia

Massimiliano Tattini

Italy

Mark Taylor

UK

Graham Teakle

UK

Keat Teoh

USA

Nancy Terrier

France

Pilar S Testillano

Spain

William Thomas

UK

John Thompson

Australia

Leeann Thornton

USA

Li Tian

USA

Zhixi Tian

China

Antonio F Tiburcio

Spain

Tina Tina

Switzerland

Henrik Tjellström

USA

Mirta Tkalec

Croatia

Per Toräng

Sweden

Pablo Tornero

Spain

Szilvia Z Toth

Georgia

Lam-Son Tran

Japan

Ben Trevaskis

Australia

Prabodh Trivedi

India

Maurizio Trovato

Italy

Mark Tucker

USA

Paul Tudzynski

Germany

Franziska Turck

Germany

Robert Turgeon

USA

Simon Turner

UK

Brett Tyler

USA

Cristobal Uauy

UK

Joshua Udall

USA

Michael Udvardi

USA

Takashi Ueda

Japan

Soledad Undurraga

USA

Turgay Unver

Turkey

Bjorn Usadel

Germany

Roland Valcke

Belgium

Alex Valentine

South Africa

Estela Valle

Argentina

Mireille Van Damme

Netherlands

Renier Van Der Hoorn

Germany

Esther Van Der Knaap

USA

Doug Van Hoewyk

USA

Klaas Van Wijk

USA

Kranthi Varala

USA

Rajeev Varshney

India

Alain Vavasseur

France

Pablo Vera

Spain

Fabio Veronesi

Italy

Paul Verslues

Taiwan

Sabina Vidal

Uruguay

Elizabeth Vierling

USA

Vikas Vikram

USA

Giovanna Visioli

Italy

Melane Alethea Vivier

South Africa

Hans Vogel

Canada

Dieter Volkmann

Germany

Albrecht Von Arnim

USA

Diter Von Wettstein

USA

Denis Vysotskii

Russian Federation

Rainer Waadt

USA

Doris Wagner

USA

Ian Wallace

USA

Michael H Walter

Germany

Yinglang Wan

China

Guixi Wang

China

Guodong Wang

China

Guoying Wang

China

Hao Wang

Hong Kong

Huanzhong Wang

USA

Jenn-Che Wang

Taiwan

Jianbo Wang

China

Junqi Wang

China

Ming Li Wang

USA

Xiaojie Wang

China

Xiaowu Wang

China

Xiyin Wang

USA

Yingxiang Wang

China

Yong Wang

China

Yuejin Wang

China

Dierk Wanke

Germany

Marilyn Warburton

USA

John Ward

USA

Thomas Warkentin

Canada

Brian Waters

USA

Chris Watkins

USA

Jim Weller

Australia

Jonathan F Wendel

USA

Elizabeth Weretilnyk

Canada

Randall Weselake

Canada

Curtis Wilkerson

USA

Joseph Williams

USA

David Willmot

USA

Roger Wise

USA

Sebastian Wolf

Germany

Marnin Wolfe

USA

Keqiang Wu

Taiwan

Liang Wu

China

Qingyu Wu

USA

Bai Xf

China

Xinli Xia

China

Xia Xianchun

China

Zhanguo Xin

USA

Yongzhong Xing

China

Ai-Sheng Xiong

China

Lin Xu

China

Yukinori Yabuta

Japan

Rattan Yadav

UK

Marna Yandeau-Nelson

USA

Chang-Hsien Yang

Taiwan

Ming Yang

USA

Zefeng Yang

China

Zhong-Nan Yang

China

Huaxun Ye

USA

Li Yi

China

Yanbin Yin

USA

John Yoder

USA

Mi-Jeong Yoo

USA

Riichiro Yoshida

Japan

Hirofumi Yoshioka

Japan

Qingyi Yu

USA

Lixing Yuan

China

Olga Yurchenko

USA

Jürgen Zeier

Germany

Shuai Zhan

USA

Jiangwei Zhang

USA

Jin-Song Zhang

China

Lizhi Zhang

USA

Peng Zhang

China

Peng Zhang

Australia

Xiaoyu Zhang

USA

Xiurong Zhang

China

Bingyu Zhao

USA

Huien Zhao

China

Nan Zhao

USA

Tianyong Zhao

China

Jun Zheng

China

Wenguang Zheng

USA

Danmeng Zhu

China

Jian-Kang Zhu

USA

Lihuang Zhu

China

Yan Zhu

China

Ying Zhu

China

Elizabeth Zimmer

USA

Laurent Zimmerli

Taiwan

Mohamed Zouine

France

Jianru Zuo

China

Eva Zyprian

Germany

